# A brief introductory review to deep generative models for civil structural health monitoring

**DOI:** 10.1007/s43503-023-00017-z

**Published:** 2023-08-23

**Authors:** Furkan Luleci, F. Necati Catbas

**Affiliations:** Department of Civil, Environmental, and Construction Engineering, University of Central Florida, Orlando, FL 32816 USA

**Keywords:** Deep generative models, Structural health monitoring, Generative adversarial networks, Diffusion models, Energy-based models, Flow-based models

## Abstract

The use of deep generative models (DGMs) such as variational autoencoders, autoregressive models, flow-based models, energy-based models, generative adversarial networks, and diffusion models has been advantageous in various disciplines due to their high data generative skills. Using DGMs has become one of the most trending research topics in Artificial Intelligence in recent years. On the other hand, the research and development endeavors in the civil structural health monitoring (SHM) area have also been very progressive owing to the increasing use of Machine Learning techniques. As such, some of the DGMs have also been used in the civil SHM field lately. This short review communication paper aims to assist researchers in the civil SHM field in understanding the fundamentals of DGMs and, consequently, to help initiate their use for current and possible future engineering applications. On this basis, this study briefly introduces the concept and mechanism of different DGMs in a comparative fashion. While preparing this short review communication, it was observed that some DGMs had not been utilized or exploited fully in the SHM area. Accordingly, some representative studies presented in the civil SHM field that use DGMs are briefly overviewed. The study also presents a short comparative discussion on DGMs, their link to the SHM, and research directions.

## Introduction

Structural health monitoring (SHM) plays a crucial role in ensuring the safety, reliability, and longevity of civil engineering structures by identifying issues at an early stage, optimizing maintenance activities, and enabling informed decision-making (Malekzadeh et al., 2015). SHM typically consists of sensing and instrumentation, data collection, preprocessing, analysis, and evaluation phases, followed by decision-making. It includes various data-driven techniques (Catbas et al., 2013, 2022).

Using SHM systems, e.g., data acquisition components, accelerometers, strain gauges, and other sensors, on every civil structure is not economical. It is widely known that data collection procedures from civil structures can be difficult and expensive, restricting the information flow obtained from structures. Due to the challenges in data collection, the data scarcity phenomenon is a crucial issue in SHM. On the other hand, information loss during the monitoring process is often caused by sensor- or transmission-based errors, making data scarcity more critical. The fact that SHM is composed of data-driven applications increases the importance of the data scarcity issue even further (Luleci & Catbas, 2022).

During the last few decades, the research and development in the civil SHM field have been very progressive due to the increasing use of machine learning (ML) and different deep learning (DL) models to address the challenging problems faced in the field, such as data scarcity (Avci et al., 2021; Luleci et al., 2022). Such models have also been used in interdisciplinary research problems (e.g., seismic damage assessment, building inventory assessment, or post-earthquake recovery models) (Soleimani-Babakamali & Zaker Esteghamati, 2022; Soleimani‐Babakamali et al., 2022; Xu et al., 2021a; Luleci & Catbas, 2023).

ML-based techniques have been a research trend for the last few decades in many SHM applications. Deep generative models, in short DGMs, are generative models with many hidden neural networks that have been highly favored in recent years across various disciplines. They are a powerful way of learning hidden data representations in data distributions and generating new data instances with variations by leveraging the flexibility of deep neural networks.

DGMs can be particularly useful in addressing the data scarcity issue in SHM. For instance, when a non-to-limited amount of labeled or high-quality data is available, DGMs can help overcome this difficulty. They could provide distinct ways to generate data for different goals. Those ways, in general, are data generation (generation only—e.g., for general data needs), lost data reconstruction (e.g., for lost or missing data points), data augmentation (e.g., for improving the class imbalance in classification problems), data domain translation (e.g., for no access to data pairs for classification problems or undamaged-to-damaged domain translation), data denoising and repairing (e.g., for noisy and bad quality data), anomaly and novelty detection (e.g., for data consists of anomalies and novelties), others (e.g., for damage identification, annotation reduction via transfer learning), and various approaches that have yet to be explored (Luleci et al., 2022). These approaches can enhance the performance, robustness, and generalization capabilities of data-driven tools used in SHM applications where data availability is non-to-limited.

## Deep generative models

Rather than creating a decision boundary in the data distribution for classification purposes, which is the discriminative approach, the generative approach aims to learn how the data distributions are shaped. The deep neural networks are used in DGMs to parametrize the generative models, increasing the model's learning capacity. When the DGMs are trained successfully, they can generate new data points similar to the data points from the unknown distribution.

DGMs generally consist of six members (Bond-Taylor et al., 2022; Ruthotto & Haber, 2021; Tomczak, 2022): autoregressive models (AMs), variational autoencoders (VAEs), flow-based models (FBMs), energy-based models (EBMs), generative adversarial networks (GANs), and lastly diffusion models (DMs). The general concepts of the DGMs are briefly explained in the subsequent paragraphs without getting into mathematics. As a side note, it was observed that while some DGMs have been explored, some others have not been studied in the SHM area during the preparation of this short review communication as of December 2022. It should also be noted that only the representative studies presented in the civil SHM field that use DGMs are briefly overviewed.

**Fig. 1:**
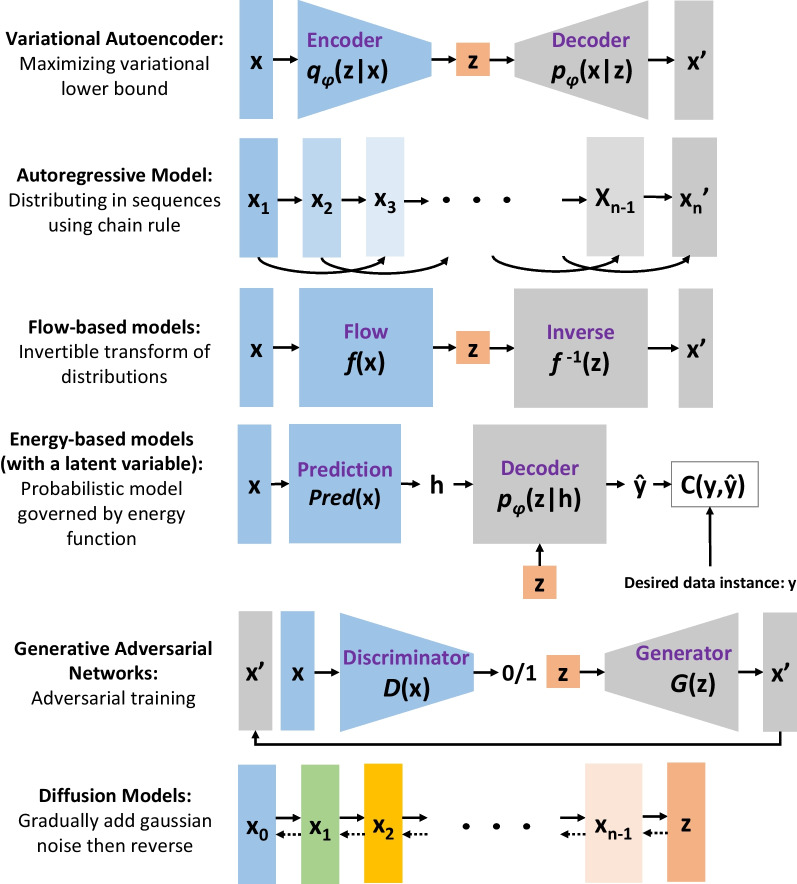
Overview of the deep generative models

Figure 1 illustrates the summary of the mechanisms of deep generative models (Chahal et al., 2020; Weng, 2021). In the figure, *x* and *x*’ are, respectively, original and synthetic data; *z* is the latent variable; *y* and $\widehat{y}$ are desired and resulted data instances; *C*(*y,*$\widehat{y}$) compares the desired and resulted instances and gives a score; *f* is the invertible transformation function; *q*_*φ*_(*z|x*) and *p*_*φ*_(*x|z*) are the probabilistic encoder and decoder; *D*(*x*) and *G*(*x*) are the discriminator and generator. While likelihood-based models such as VAEs, AMs, FBMs, EBMs, and DMs can be trained stably, training implicit models like GANs can be unstable. In VAE, only the lower bound is provided, and the likelihood function cannot be precisely computed, which is also true for EBMs requiring calculating the partition function. AMs suffer from the sampling process, which makes the inference extremely slow due to the autoregressive manner of generating new data points; however, they are one of the most efficient likelihood models in terms of their structure. EBMs and DMs require to run Monte Carlo for inference, slowing down the generation. Nevertheless, DMs are currently state-of-the-art DGM, demonstrating better generative performance than even GANs (Dhariwal & Nichol, 2021).

### *Variational Autoencoders *(*VAEs*)

VAEs were first introduced by Kingma and Welling (2013), which are probabilistic generative models that combine the concepts of autoencoders and variational inference. Autoencoders are neural network architectures that learn to encode and decode data, compressing it into a lower-dimensional latent space. Variational inference is a statistical technique used to approximate complex probability distributions. VAEs introduced a new approach to unsupervised learning by leveraging the power of neural networks and variational inference. The key innovation was introducing a latent variable model with a well-defined probabilistic interpretation. VAEs enable efficient encoding of data and generation of new samples by sampling from the latent space. The training process of VAEs involves optimizing two objectives: the reconstruction loss, which ensures the faithful reconstruction of input data, and the Kullback–Leibler (KL) divergence, which encourages the latent space to follow a prior distribution, typically a multivariate Gaussian. Since their introduction, VAEs have gained significant attention and found numerous applications. They have been utilized in tasks such as image synthesis, anomaly detection, data generation, and representation learning. Researchers have explored various architectural modifications and training techniques to improve the quality of generated samples and address challenges like posterior collapse. The development of VAEs has opened up new possibilities in generative modeling and probabilistic inference, providing a versatile framework for learning and manipulating complex data distributions.

**Fig. 2:**
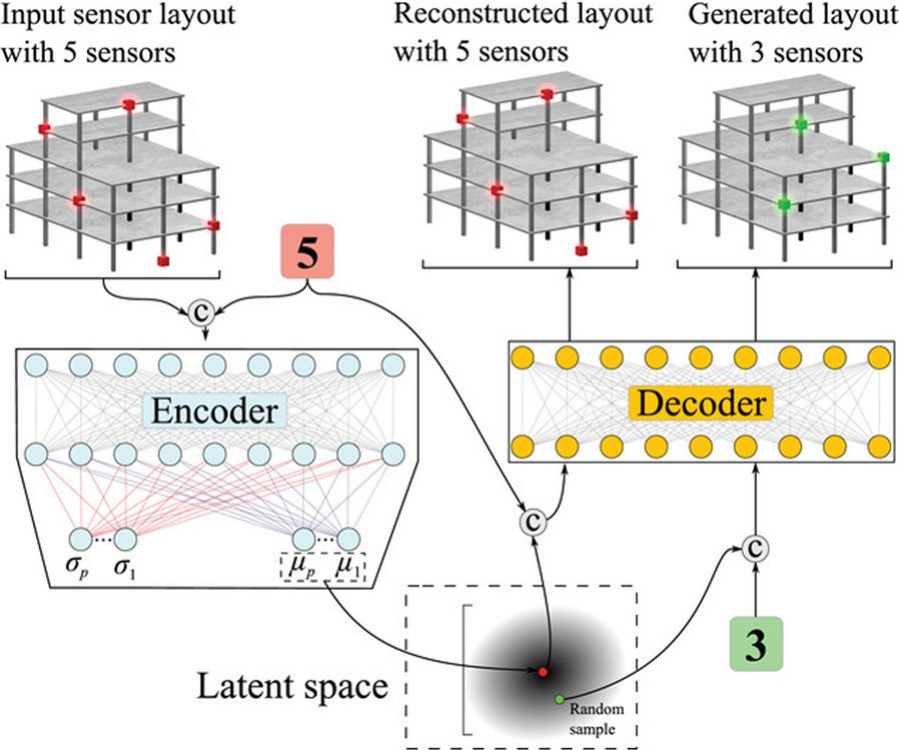
Generating sensor placement layouts with a particular number of sensors using a conditional variational autoencoder, where the introduced methodology is tested on a nine-story reinforced concrete moment frame (Sajedi & Liang, 2022)

Leveraging the probabilistic approach integrated with an autoencoder helps VAEs to achieve great data generation performances compared to vanilla autoencoders (Kingma & Welling, 2019; Mayank Mittal & Harkirat Singh Behl, 2018). VAEs are also often compared with GANs in terms of their generation performances. The use of VAEs in SHM can go back to the early 2020s (Liu et al., 2019b; Ma et al., 2020), presenting anomaly detection on railways and feature extraction via VAE. Since then, several studies have been presented employing the generative skill of VAEs in civil SHM for various purposes, such as damage and anomaly identification, and condition assessment (Anaissi et al., 2023; Pollastro et al., 2022; Xu et al., 2021b; Yuan et al., 2021; Zhou et al., 2022), and optimal sensor placement (Sajedi & Liang, 2022) (Fig. 2), addressing data scarcity challenge in the SHM domain in one way or another.

### Autoregressive models (AMs)

AMs have a history rooted in time series analysis and evolved with ML advancements. They gained prominence with autoregressive moving average (ARMA) models in the 1950s, which captured dependencies in time series data (Box, 1970). In the 1980s, AMs were applied to speech and audio processing, enabling the synthesis of realistic speech (Gray, 2010). The introduction of restricted Boltzmann machines in 2006 facilitated efficient training of autoregressive models. Notably, the development of deep AMs and PixelRNN (Oord et al., 2016a, 2016b) demonstrated the potential of DL in modeling pixel dependencies in images. The subsequent introduction of WaveNet (Oord et al., 2016a, 2016b) revolutionized AMs for speech and audio generation. Inspired by the transformer architecture (Vaswani et al., 2017), transformer-based autoregressive models further expanded AM capabilities of AMs across various domains. AMs continue to advance, leveraging DL techniques to model sequential dependencies and generate highly realistic and diverse samples.

**Fig. 3:**
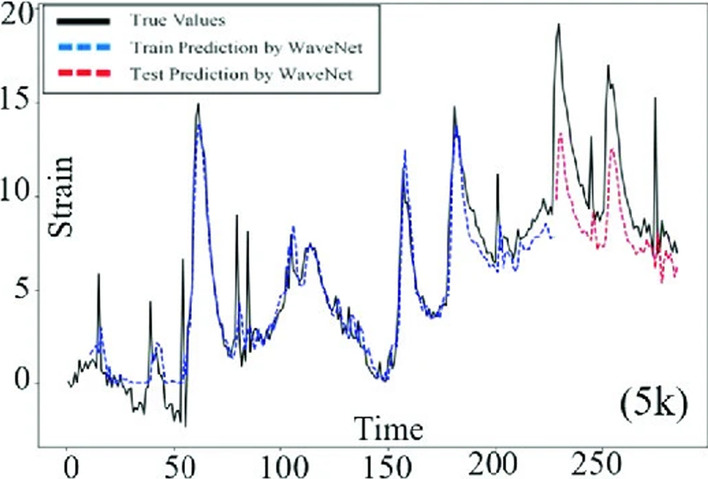
The ground truth strain response values collected from a bridge structure (black), the estimated strain train data (blue), and the estimated strain test data (red) using WaveNet (Psathas et al., 2022)

AMs implicitly determine a distribution over sequences by using the chain rule for conditional probability. In this sequence, each step in the distribution is predicted based on the previous steps. Basically, AMs take the previous data in a sequence to predict a future value in that sequence. Thus, AMs are generally a better fit for time series with an intrinsic sequence of time steps, where they truly excel. One of the best-known models is WaveNet for audio generation (Oord et al., 2016a, 2016b). AMs are also used for images using sequential models for the pixels, such as the PixelRNN model (Oord et al., 2016a, 2016b) but are not great at image generation. Among other DGMs, it is essential to note that AMs are sequential but are still feedforward. Additionally, while they are generative, they still use a supervised approach. These facts make AMs faster, more stable in training (but very slow in data sampling and have poor scaling properties), and more straightforward and intuitive than the other DGMs. In civil SHM, AMs have been quite popular among researchers for years, and they were mainly used for feature extraction for damage identification using ARMA or variants (Entezami et al., 2021; Gul & Catbas, 2009; Liu et al., 2019a; Rajeev et al., 2022). AMs are also used for future data estimation (Psathas et al., 2022) (Fig. 3); however, feature extraction purposes have been seen more often in the literature. Figure 3 shows the estimated strain data from the train passage using WaveNet (Psathas et al., 2022).

### *Flow-based models *(*FBMs*)

Flow-based models have emerged as a powerful class of generative models in recent years. The history of flow-based models can be traced back to the early 2010s (Dinh et al., 2014) when the concept of normalizing flows was introduced. Normalizing flows aim to model complex probability distributions by transforming simple distributions through a series of invertible mappings. In 2015, Dinh et al. proposed the Real NVP (real-valued non-volume preserving) architecture (Dinh et al., 2016), which allowed for flexible and tractable transformations in high-dimensional spaces. This marked a significant advancement in flow-based modeling. Subsequently, other flow-based architectures like Glow (Kingma & Dhariwal, 2018), FFJORD (Grathwohl et al., 2018), and Neural Spline Flows (Durkan et al., 2019) were introduced, further improving the expressiveness and scalability of flow models. FBMs have gained attention due to their ability to model complex data distributions, efficient sampling, and exact likelihood evaluation.

VAEs and GANs do not explicitly learn the probability density of real data, and they are intractable. FBMs (Danilo Jimenez Rezende & Shakir Mohamed, 2015) tackle this challenge by modeling a probability distribution using normalizing flows, a statistical tool for density estimation. In other words, FBMs learn the probability density explicitly, which makes them tractable. FBMs being tractable also makes the objective of the training simply the negative log-likelihood. Normalizing flows assist FBMs in modeling for a better distribution approximation leveraging the change-of-variable theorem of probabilities for transforming a distribution into a complex one. This is achieved by implementing a sequence of invertible transformation functions. The variables are repeatedly substituted for a new one based on the change-of-variable theorem to obtain a probability distribution of the end target variable. Essentially, FBMs are constructed by a sequence of invertible transformations with the aid of normalizing flows. Some notable FBMs are available in these references (Dinh et al., 2015; Kingma & Dhariwal, 2018). In addition, more recently, the normalizing flows were incorporated into a Diffusion Model (Qinsheng Zhang & Yongxin Chen, 2021). To the best of the authors' knowledge, the use of FBMs for SHM has not been observed during the preparation of this manuscript.

### *Energy-based models *(*EBMs*)

Energy-based models (EBMs) have a long history in ML and have undergone several developments. The concept of EBMs can be traced back to the 1980s, and since then, the EBMs have been improved and extended (LeCun et al., 2006). When they were first introduced as a framework for unsupervised learning (Ackley et al., 1985; Hopfield, 1982), the Boltzmann machines were an early form of EBMs that employed the notion of energy to model joint probability distributions. However, training Boltzmann machines were computationally challenging. In recent years, the development of DL techniques and advancements in optimization algorithms have revitalized the interest in EBMs. Researchers have explored novel architectures, such as GANs and score-based models, to improve the learning and generation capabilities of EBMs. Ongoing research continues to refine and expand the applications of EBMs, making them promising tools for generative modeling, representation learning, and anomaly detection.

EBMs are a probabilistic model controlled by an energy function that defines the probability of a particular state. Essentially, they capture data dependencies by applying a probability scalar “energy” (a measure of compatibility) to each configuration of the variables. In that regard, inference includes setting the value of observed variables to 1 and then identifying the values of the rest of the variables that minimize that scalar energy amount. The learning can be accomplished by obtaining an energy function that correlates low energies with correct values of the rest of the variables and higher energies with incorrect values. EBMs use a unified framework combining all the probabilistic and non-probabilistic approaches for learning, especially for training graphical and structured models. The challenge of estimating normalization constant in probabilistic models does not exist in EBMs, which allows for more flexibility in the design of the learning process. However, EBMs suffer from modeling high-dimensional data. Although EBMs have been a research field for several decades, including some recent studies (Shuangfei Zhai & Cheng, 2016; Yilun Du & Igor Mordatch, 2019; Zhao et al., 2017), no studies are observed using EBMs in the civil SHM field, again to the best knowledge of the authors at this time.

### *Generative adversarial networks *(*GANs*)

**Fig. 4:**
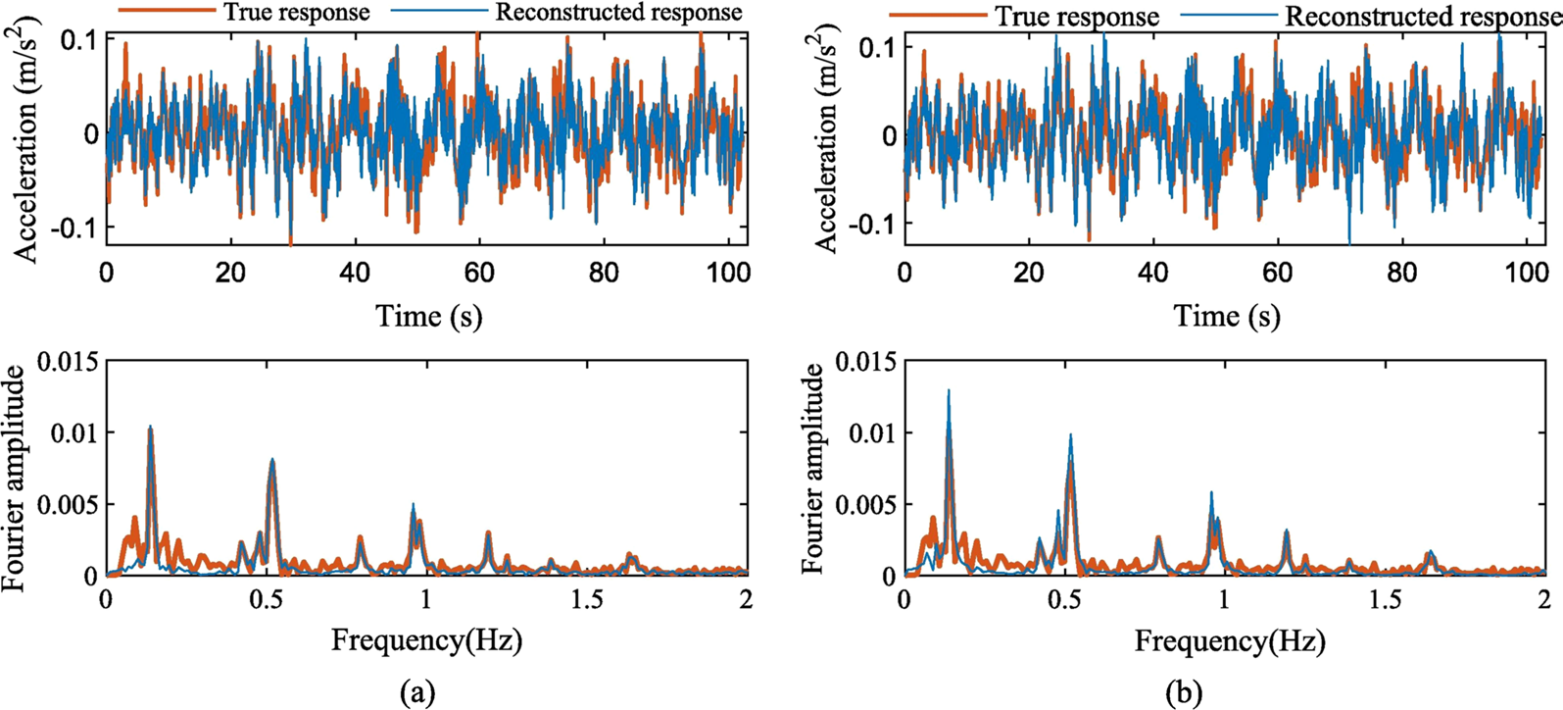
**a** The original and reconstructed acceleration responses in the time and frequency domain using self-attention mechanism enhanced generative adversarial network; **b** The original and reconstructed acceleration responses in the time and frequency domain using segment-based generative adversarial networks (Fan et al., 2023)

When GAN was first released in 2014 (Goodfellow et al., 2014), it received significant attention due to its novel approach (adversarial training concept – minimax game) and cutting-edge performance in image generation. GAN contains two networks: a generative network and a discriminator network. Essentially, the generator learns to generate similar data samples to the real dataset based on the discriminator’s output, while the discriminator also learns about the real data domain. In other words, both networks attempt to overcome each other in a minimax game; while the generator tries to fool the discriminator with the generated images, the discriminator tries to predict the synthetic and real images. Followed by its release, many researchers focused on improving the training of GAN due to its well-known unstable and no-convergence training process and mode collapse (less diversity in generated outputs) (Goodfellow, 2016; Salimans et al., 2016), WGAN (Arjovsky et al., 2017), WGAN-GP (Gulrajani et al., 2017). Moreover, there are many notable works using GANs, such as CycleGAN (Zhu et al., 2017), StyleGAN (Karras et al., 2018), and ESRGAN (Wang et al., 2018). Using GANs (original GAN and variants) for civil SHM applications is a popular research activity, and they were found beneficial for several challenges in SHM. For instance, lost data reconstruction (Fan et al., 2023; Jiang et al., 2022; Lei et al., 2021), data augmentation (Luleci et al., 2021), data domain translation (Luleci et al., 2023a, 2023b), anomaly and novelty detection (Soleimani‐Babakamali et al., 2022), and (Wang et al., 2019) data denoising. Figure 4 presents the use of GAN for true and reconstructed sensor data instances in their respective time and frequency domains. GANs were considered state-of-the-art generative models by many in terms of the quality of their generative performances until the recent rise of Diffusion Models (DMs).

### *Diffusion models *(*DMs*)

The history of DMs can be traced back to 2015, inspired by non-equilibrium thermodynamics (Sohl-Dickstein et al., 2015), aiming to develop a learning approach that achieves analytical flexibility and tractability. Diffusion models, also known as denoising diffusion probabilistic models (DDPMs), have gained prominence in the field of generative modeling in recent years. These models employ a different approach compared to traditional generative models by explicitly modeling the process of iterative denoising a corrupted input to generate realistic samples.

The essential concept of DM is to successively add random noise to the data (image) through a Markov chain sequence to eventually obtain an isotropic Gaussian noise. Then, learn to reverse the forward diffusion process via backward propagation to reconstruct (or denoise) the desired data from the Gaussian noise. Some of the major differences of DMs between and the other DGMs are being able to generate highly realistic images and yield more diversity even better than GANs, having stable training procedures, and being able to be conditioned on a wide variety of inputs (Dhariwal & Nichol, 2021; Ho et al., 2020; Song & Ermon, 2019). One other unique property of DMs is that the latent space has the same dimensionality as the original data, which benefits DMs in terms of less computation. More recently, DMs have also shown remarkable success in the image and video generation, such as Imagen (Saharia et al., 2022) and Imagen Video (Ho et al., 2022) from Google, Dall-E 2 (Ramesh et al., 2022) from OpenAI and Make-A-Video (Singer et al., 2022) from Meta. Since DMs are a new research area in the Artificial Intelligence field, no study seems to have been in the literature using Diffusion Models in the SHM domain.

## Discussion: DGMs and future directions in SHM

While each DGM has its drawbacks, some can be useful in civil SHM applications. VAEs enable efficient sampling from the latent space and can learn meaningful latent representations. However, they may produce blurry samples and struggle with capturing complex data distributions.

AMs are a good choice for time series-based applications, yet suffer from relatively slow inference and low QDS, unlike GANs. AMs can be computationally efficient but may struggle with capturing complex dependencies and generating high-dimensional data.

FBMs are much easier to converge and more stable during training, unlike VAEs and GANs. They provide exact likelihood estimation, enabling efficient density estimation and sampling. However, they can be computationally expensive during training and may struggle with modeling complex distributions.

EBMs show great out-of-distribution generalization skills thanks to their penalization learning strategy via the scalar energy values, making them a good candidate for knowledge transfer applications between dissimilar civil structures (Luleci & Catbas, 2022). EBMs can model complex data distributions and handle missing data, but they can be challenging to train and require sophisticated techniques for efficient inference.

Through adversarial training, GANs learn to generate high-quality and diverse samples. They have shown remarkable results in generating realistic samples but can be challenging to train and suffer from mode collapse, making training convergence difficult.

DMs reach state-of-the-art data generation performance; training them is easier and more stable, they are more explainable, and can be a better fit for time series due to their chain sequence approach. DMs provide a tractable likelihood estimation, making them suitable for density estimation tasks. They have shown promising results in generating high-quality samples but can be computationally expensive in sampling due to the iterative nature of the diffusion process.

Overall, these generative models have different strengths and limitations. The choice of model to use depends on the specific task, data characteristics, and trade-offs between sample quality, training stability, and computational efficiency, as presented in Table 1. From the previous paragraphs and Table 1, one can deduce that DMs should be the top-choice generative models. While this might be true, each DGM has its strengths and limitations. For instance, although DMs perform the best generative skills, the sampling speed is relatively slow compared to the other DGMs, forcing researchers to improve this limitation (Ulhaq et al., 2022). On the other hand, some other researchers combine the best of each DGM, such as training GANs with Diffusion (Wang et al., 2022), where the sampling speed is significantly improved. Another example is taking advantage of the strengths of normalizing flows and diffusion (Qinsheng Zhang & Yongxin Chen, 2021) (diffusion normalizing flow) to improve the training and sampling speeds of FBMs and DMs while enhancing the generation quality.

**Table 1:** Summary of describing each DGM in training and sampling speed, efficiency in parameter, sample quality-diversity-scalability (QDS), the tractability of density, the highlights, and some of their applications

	Variational autoencoders	Autoregressive models	Flow-based models	Energy-based models	Generative adversarial networks	Diffusion models
Training speed	Moderate	Moderate	Low	Moderate	High	High
Sampling speed	Moderate	Low	High	Low	High	Low
Param. Effic	Moderate	Low	High	Moderate	High	High
Sample QDS	Moderate	Low	Moderate	Moderate	High	High
Tractability of exact density	Partially intractable (approximates the density)	Tractable	Tractable	Intractable	Intractable	Tractable
Highlight	Great at sampling, though sample quality could be bad if the posterior collapses	Extremely slow sampling process but constitute one of the best likelihood-based models	Very stable and easier convergence in training, but sample quality can be bad	Very flexible but require monte-carlo; thus, they are computationally expensive	Though very high QDS, they are hard to train; thus, training convergence is difficult	State-of-the-art QDS and more explainable than GANs, though slow sampling
Some of the applications^*^	Anomaly detection (Zhou et al., 2022), damage identification (Ma et al., 2020; Pollastro et al., 2022), and optimal sensor placement (Sajedi & Liang, 2022)	Feature extraction for damage identification (Entezami et al., 2021; Gul & Catbas, 2009; Rajeev et al., 2022) and future data estimation (Psathas et al., 2022)	No study is observed as of conducting this study	No study is observed as of conducting this study	Data augmentation (Luleci et al., 2023c), lost data reconstruction (Fan et al., 2023; Lei et al., 2021), domain translation (Luleci et al., 2023a), for more info, see GAN in SHM lit. rev. (Luleci et al., 2022)	No study is observed as of conducting this study

Note that this study does not present an extensive literature review, and only some representative studies that use DGMs are referenced. Also, although each DGM has its drawback, several models exist in the literature using multiple DGMs together to complete their drawbacks, such as Diff-GAN (Diffusion Models and Generative Adversarial Networks) (Wang et al., 2022)

In general, DGMs could provide distinct ways to generate data for different goals, as mentioned in the introduction, such as:

- *Data generation *(*only*) to generate data for general needs;
- *Lost data reconstruction* to recover the lost or missing data due to SHM sensorial or transmission errors;
- *Data augmentation* to improve the low performance in damage identification applications due to class imbalance of the training dataset;
- *Data domain translation* to enable access to the paired data points for the latter damage identification applications;
- *Anomaly and novelty detection* to identify anomalies, novelties, and outliers in structural response measurements, which could indicate potential issues with the structure;
- *Data denoising, deblurring, and repair* to remove noise, blur, and enhance the quality of data;
- *Others*, such as damage identification, annotation reduction via transfer learning to reduce data labeling for classification applications, or generating sensor placement layouts.

**Fig. 5:**
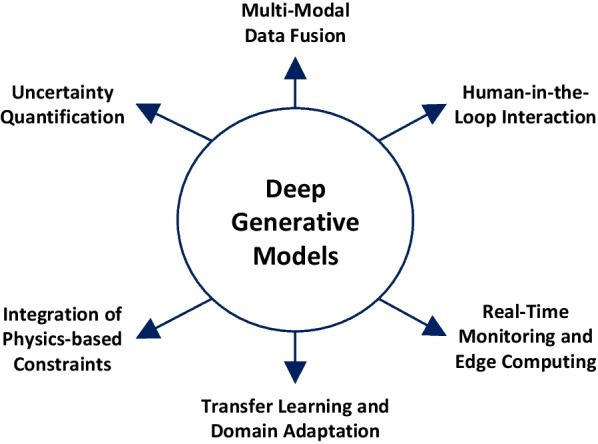
Some of the research directions for using DGMs in the SHM field

Future research directions for using DGMs in SHM could include several ways in which some are already being explored (Fig. 5).

*Transfer learning and domain adaptation:* Developing techniques for transferring knowledge and models from one structure to another or adapting pre-trained models to new structures. This includes adapting pre-trained models to new structures, leveraging domain knowledge, or simply building novel models for efficient generalization to other domains. This would facilitate the deployment of DGMs in real-world SHM scenarios where labeled data may be limited or unavailable, addressing the data scarcity issue.

*Real-time monitoring and edge computing:* Investigating the implementation of DGMs on edge devices or within the infrastructure of the monitored structures. This would enable real-time monitoring, analysis, and decision-making, reducing the dependence on cloud-based processing and enhancing the scalability and efficiency of SHM systems. For instance, in the event of lost data during monitoring, DGMs could assist in reconstructing the missing part in real time.

*Integration of physics-based constraints:* Investigating approaches incorporating physics-based constraints and structural mechanics principles into DGMs. This includes developing models that learn from data and capture the underlying physical behavior of structures, improving the accuracy and reliability of the SHM data or other SHM model generations.

*Uncertainty quantification:* Investigating methods to incorporate uncertainty estimation in DGMs for SHM. This includes developing probabilistic models that can provide confidence intervals or probability distributions for anomaly detection and damage assessment tasks, e.g., generating probability distributions for certain uncertainty ranges for different operational scenarios and structures.

*Multi-modal data fusion:* Exploring approaches to fuse data from multiple sensors and modalities using DGMs. This involves integrating different types of sensor data, such as vibration, strain, and temperature, to improve anomaly detection and provide a comprehensive understanding of structural health. For instance, a DGM could be used to generate temperature- and humidity-induced vibration data, providing a more holistic view of the operational status of the structure.

*Human-in-the-loop interaction:* Exploring interactive approaches that involve human experts in the loop to guide and refine the generative models' outputs. This would leverage the expertise and domain knowledge of human operators to enhance interpretability and reliability and supports decision-making processes in SHM applications.

DGMs also could be trained on structural response datasets for loading conditions like wind, earthquakes, or floods. These models could then be used to generate structural responses for different scenarios by the end-user for producing varying structural behavior simulations of how a structure is likely to respond to different types of loading, allowing engineers to understand the behavior of the structure better and identify potential areas of weakness. This could help engineers prioritize repairs and maintenance as well as take preventative measures to avoid catastrophic failures.

These future research directions aim to advance the use of DGMs in SHM by addressing key challenges, improving model performance, and facilitating their practical deployment in real-world structural monitoring scenarios.

## Conclusion

The research and development in the civil SHM domain have been very progressive for the last few decades due to the increasing use of ML to tackle the challenging problems faced in the field (Avci et al., 2021; Azimi et al., 2020; Bao & Li, 2021). On the other hand, using deep generative models (DGMs) has also been a trend across many disciplines lately, demonstrating very efficient solutions for particular applications. Civil SHM is one of these disciplines that researchers have just begun exploring to use some members of DGMs towards SHM applications.

It is important to note that data scarcity is a significant challenge in civil SHM due to data collection tasks from civil structures being challenging. While data collection from every civil structure is not economically feasible, a large portion of the structures is worth monitoring due to the growing concern for the better management, operation, and safety of civil structures. Even when a few are monitored, SHM system-based (sensor or transmission errors) are typical, resulting in sensorial data loss. The fact that SHM applications, such as damage diagnosis and prognosis, rely on data-driven solutions makes the challenge of data scarcity even more significant. Therefore, employing deep generative models (DGMs) for SHM applications is critical, considering their excellent data generation performances as demonstrated in the literature.

While no studies exist using FBMs, EBMs, and DMs based on the literature review as of December 2022, quite a few works are available using GANs in the civil SHM domain. On the other hand, AMs are primarily used for feature extraction for damage identification, future data estimation, and similar applications. Lastly, several studies use VAEs in civil SHM for various purposes, such as anomaly detection, damage identification, and optimal sensor placement.

It can be argued that there is a large room for research and development using DGMs for data generative-based applications in the civil SHM field, particularly with case studies.

## Data Availability

Not applicable.
